# Understanding Psychologists’ Usage, Knowledge, and Attitudes Toward Digital Mental Health Solutions for Refugees and Migrants: Exploratory Cross-Sectional Survey in Sweden

**DOI:** 10.2196/75263

**Published:** 2026-03-03

**Authors:** Kristofer Vernmark, Anahita Geranmayeh, Naira Topooco, Gerhard Andersson, Shervin Shahnavaz

**Affiliations:** 1 Department of Behavioural Sciences and Learning Linköping University Linköping Sweden; 2 Centre for Psychiatry Research Department of Clinical Neuroscience Karolinska Institutet, & Stockholm Health Care Services Stockholm, Region Stockholm Sweden; 3 Department of Psychology Uppsala University Uppsala Sweden; 4 Department of Biomedical and Clinical Sciences Linköping University Linköping Sweden; 5 Department of Clinical Neuroscience Karolinska Institutet Stockholm Sweden

**Keywords:** digital mental health, internet-based, ICBT, psychologist, stakeholder, refugee, migrant, implementation, health care, knowledge, attitudes

## Abstract

**Background:**

The rising number of refugees and migrants has created growing mental health needs that health care systems struggle to address. Providing assessment and treatment for mental health problems in a digital format could help increase access to care and facilitate the provision of adapted interventions. Psychologists are key stakeholders in the delivery and influence of clinical services within routine care settings, but there are limited data on their perspectives regarding the use of digital solutions to assess and treat common mental health problems in refugees and migrants.

**Objective:**

This study aimed to examine psychologists’ usage, knowledge, and attitudes toward digital mental health solutions for assessing and treating common mental health problems in refugees and migrants within the Swedish health care system.

**Methods:**

A cross-sectional online survey was conducted among psychologists in Sweden between December 2023 and February 2024. Responses included Likert-scale items and categorical variables, which were analyzed using descriptive statistics, independent samples *t* tests, and Fisher exact test to explore differences between subgroups.

**Results:**

A total of 81 psychologists responded to the survey. Among them, 58 (72%) were women, and nearly half (40/81, 49%) worked in a public health care region. Respondents showed the highest acceptance for guided internet-based cognitive behavioral therapy (ICBT), blended treatment, and videoconferencing therapy. Only 20% (16/81) reported using digital solutions for refugees or migrants with mental health problems. Most respondents had low or very low knowledge of digital assessment and screening (61/81, 75%) and digital treatment (58/81, 72%) for these groups. Those using digital formats for refugees and migrants, or working in a setting that did so, had significantly higher ratings on all 5 knowledge items compared to those that did not (*P<*.001 to *P*=.01). Respondents emphasized the importance of digital solutions being provided in refugees’ and migrants’ native languages (70/81, 86%) and being culturally adapted (56/81, 69%). Those using digital formats for refugees and migrants considered cultural adaptation less necessary (*P*=.05). The preferred implementation approach was through specialized or decentralized units in primary care (66/81, 81%).

**Conclusions:**

While psychologists recognize the potential of digital mental health solutions, significant barriers remain, including limited knowledge and experience with using digital formats for refugees and migrants. Psychologists prefer digital solutions in the native language of refugees and migrants that are implemented at the primary care level. The need for cultural adaptation should be further explored. Addressing psychologists’ preferences could facilitate the future integration and implementation of digital formats for refugees and migrants in routine care settings.

## Introduction

The world is experiencing an unprecedented rise in the number of forcibly displaced people. By the end of 2024, over 123 million people across the globe were displaced or stateless, having left their homes due to war and conflict, natural disasters, consequences of climate change, or political changes, including 31 million refugees [1]. Refugees often endure pre-, peri-, and postmigration challenges that increase the risk of developing mental health problems [2] and complicate integration in the new host country [3]. Research in Swedish and international contexts shows that refugees and certain migrant groups experience elevated symptoms of depression, anxiety, and posttraumatic stress [4-6] and that these problems are also prevalent among children and adolescents [7]. Although effective treatment options exist for these conditions [8], the use of health care services among refugees and migrants differs from that of the native-born population. Refugees and migrants tend to underuse available resources and experience barriers to care due to cultural differences such as language difficulties, lack of awareness, and mental health stigma [9-12], leading to untreated mental health problems and further societal marginalization and exclusion from health care systems.

One way of increasing the reach of mental health interventions and overcoming the lack of access and availability of mental health professionals [13] is by providing digital psychological treatment. This format of delivery has expanded considerably during the last 2 decades [14,15] and was further accelerated during the COVID-19 pandemic [16,17]. Internet-based treatment and videoconferencing therapy have been found effective for common mental health problems, including social phobia, generalized anxiety, panic disorder, posttraumatic stress disorder, and depression [18,19], yielding similar results as face-to-face treatment [20,21]. Other digital formats, such as chat, email therapy, and smartphone apps, are being provided by mental health care professionals and have shown promising results [22-25]. Also, the use of digital material together with face-to-face sessions in a blended format, a combination often preferred by providers [26], has been shown to be effective for common mental health problems [27]. Modern information technology is also used for the assessment of mental health status. Providing questionnaires and screening measures online has been shown to be equivalent to paper and pencil provision [28] and can be used to screen for refugee mental health problems [29]. Digital mental health interventions have been shown to be less effective for culturally and linguistically diverse groups such as ethnic minorities, indigenous communities, migrants, and refugees [30,31]. Few studies so far have examined digital solutions tailored to the specific needs of refugees and migrants [32-34], even though there exist specific frameworks for the cultural adaptation process [35], studies describing the adaptation process [36], and research suggesting that culturally adapting psychological treatment can increase both compliance and effect [37]. There is also a need to learn more on how digital interventions can be scaled and integrated into health care systems in different settings, including factors related to acceptability, practice, and structure [38].

When introducing and implementing a new method, the influence of health care professionals as stakeholders is crucial and considered an important determinant in implementation frameworks [39]. As implementation is essentially a social process, the views of health care professionals also exert a strong influence on dissemination efforts [40,41] and they have been shown to be gatekeepers that strongly influence the implementation of internet-delivered treatment in routine mental health care settings [42]. Concerning mental health services, psychologists constitute an important health care profession often engaged in implementing and providing new services [43]. In Sweden, the term “Psychologist” is a protected title, and psychologists are key stakeholders, as they play an important role in the health care system, assessing and treating common mental health problems in primary care and providing interventions for more severe problems in specialized settings specifically targeting refugee health problems. The psychology profession has undergone a digital transition within the Swedish health care system in private and public settings, both before [44] and during the COVID-19 pandemic [45], with many already using digital modalities for assessment and treatment in their everyday work [15,46]. The opinions of therapists, including psychologists, in this transition have generally been positive [44,47]. However, far less is known about psychologists’ knowledge and attitudes toward the use of digital mental health solutions for refugees and migrants. Sweden experienced a significant increase in the number of refugees and asylum seekers during the 2010s, peaking in 2015, primarily from Arabic-, Farsi-, and Dari-speaking countries such as Syria, Iraq, Iran, and Afghanistan [48], Today, Arabic is the second most commonly spoken language in Sweden [49], and migrants and refugees with insufficient Swedish proficiency often cannot access mental health treatment without interpreter support or the use of translated material. Therefore, there is a specific need to learn more about psychologists’ perspectives of providing digital interventions for refugees and migrants not fluent in the Swedish language.

The objective of the survey was to explore psychologists’ use, knowledge, and attitudes toward digital solutions for the assessment and treatment of common mental health problems in refugees and migrants with limited proficiency in Swedish.

## Methods

### Study Context

The survey was conducted as part of the “SAHA” (“health” in Arabic) project, an academic collaboration aimed at developing and evaluating digital assessments and interventions for refugees and migrants speaking Arabic, Dari, or Farsi [50].

### Study Procedure and Participants

The Checklist for Reporting Results of Internet E-Surveys (CHERRIES) was used to guide the reporting of the survey [51]. A filled-out version of the checklist can be found in Multimedia Appendix 1. An online self-report survey was distributed between December 2023 and February 2024. A convenience sampling method was used to collect the data. Survey respondents were licensed psychologists working mainly in private, public, or nongovernmental settings in Sweden. The survey intentionally targeted both psychologists working with refugees and migrants and those without such experience but working with interventions for common mental health problems to explore potential differences in responses based on workplace and experience with the target groups. Study information and an invitation to participate in the survey were distributed using a variety of digital channels of communication available for the research group. This included emails to selected individual representatives from a private psychology consultant company, a private health care provider, 7 public health care settings, 2 nongovernmental organizations (NGOs), and 2 professional interest groups for psychologists. Selected representatives received information about the study to distribute within their organizations. Recruitment also included distributing information about the study through mailing lists for employees at 2 public research and clinical centers, one mailing list for cognitive behavioral therapy therapists, and social media posts in 6 closed professional Facebook (Meta Platforms) groups for psychologists (≈200-10,000 members). Data on actual reach through these different distribution channels are not available. Posts and emails described the study as a survey for psychologists about their perspectives on using digital solutions for mental health problems in refugees and migrants and included a link to the online survey. Reminders were sent once or twice through the above-mentioned distribution channels. Invitations included a brief description of the study, the intended respondents, and a link to the survey (see Multimedia Appendix 2).

### Survey Development

The authors KV and AG developed the initial version of the survey, drawing on a previous study by the research group at Linköping University that examined knowledge, attitudes, acceptability, and near-future expectations regarding digital treatment formats for depression [26]. Questions considering the provision of care were added to further address implementation perspectives. The new survey was reviewed by all researchers within the SAHA project who had expertise in digital assessment and treatment, refugee and migrant groups, and cultural adaptations. Based on the team review, changes were made concerning terminology, comprehensibility, the inclusion of topics related to the research project, themes regarding refugees and digital mental health, and alignment with overarching analytic entities such as knowledge and attitudes. A beta version of the survey was distributed again to the SAHA research group, as well as 3 psychologists representing the public, private, and nongovernmental (NGO) sectors, to test for contextual fit. Based on this feedback, the survey was again modified, including adjustments of important terminology, grammatical errors, and clarification of how questions were related to the purpose of the study, until a final version of the survey named SAHA-Stakeholder was decided upon. The terms “migrant” and “refugee” were defined at the beginning of the survey for respondents and were used consistently throughout. As phrased, these terms included individuals with no or limited proficiency in Swedish and did not specify current residence status (eg, individuals with no residence permit, asylum seekers, or holders of temporary or permanent permits).

The final version of the survey included 26 closed questions and 5 open-ended free text questions. It was distributed through the Iterapi platform (Linköping University, Sweden), a secure and widely used solution for providing online surveys, questionnaires, and internet-based interventions [52], and took approximately 15-20 minutes to complete. Two of the open-ended questions, addressing specific barriers and facilitators to implementation, were analyzed separately using conventional content analysis and will be reported in a separate manuscript. Closed questions were conveyed as binary questions (yes or no), nominal scale alternatives (eg, children, youth and adolescents, adults, and older adults), or multiple-choice selections. All knowledge questions used 5-point Likert scale answers, ranging from 1 (very low) to 5 (very high). Three of the closed questions included an option for the respondent to provide a free-text answer if the available answers did not apply to the respondent. Data on ethnicity were not collected due to restrictions on this type of data collection in Sweden. The survey was developed and distributed in Swedish. For a translated English version of the survey and a detailed description of the Iterapi platform, see Multimedia Appendices 3 and 4.

### Data Analysis

All quantitative data were processed and analyzed using descriptive and inferential statistical procedures in IBM SPSS Statistics (version 29.0; IBM Corp). All inferential statistics were considered exploratory due to the limited sample size and the absence of an a priori power analysis. Responses to the survey questions on respondent characteristics from the survey questions (items 1-8, 12, and 13) were recoded into binary variables to ensure adequate cell sizes in correlational analyses and *t* tests, thereby supporting valid statistical comparisons and improving the interpretability of results. Details of the recategorization are provided in Multimedia Appendix 5.

Due to the low expected frequencies in some cells, Fisher exact test was used as an alternative to the chi-square test for analyzing nominal and ordinal data. Moreover, adjusted standardized residuals were examined to assess the impact of each cell in analyses exceeding the 2x2 table format, with a value of 1.96 indicating significant deviations from expected frequencies. Since this analysis provides descriptive indicators of cell contributions rather than effect size measures, and most tables exceeded the 2x2 format, odds ratios and confidence intervals were not calculated.

Two-tailed *t* tests were used to analyze Likert-scale responses to the 5 knowledge questions and compare groups using the binary categories. The data were treated as continuous variables, in line with the common use of parametric tests for Likert-type scales in social science research [53-55]. The Shapiro-Wilk test results indicated that the data were not normally distributed. However, *t* tests are generally considered robust to such violations, based on the central limit theorem, when sample sizes are well above 30, such as in this study. To assess the robustness of the findings given these deviations, a nonparametric alternative—the Mann-Whitney *U* test—was conducted as a sensitivity analysis. The results were consistent with those of the independent samples *t* test, with one exception. Free-text responses from the 3 open-ended questions on currently used digital tools, preferred digital tools, and gatekeepers, as well as from the 3 “Other” response options, were grouped into categories but not formally analyzed. They are included in the discussion section only to provide contextual understanding.

### Ethical Considerations

Ethical approval was obtained from the Swedish Ethical Review Authority (2022-04274-02). All participants provided informed consent digitally before accessing the survey. Participation was voluntary, and respondents could not proceed to the questionnaire without providing consent (see Multimedia Appendix 6). Participants were informed about the purpose of the study, the voluntary nature of participation, their right to withdraw at any time prior to submission, and how their data would be used. Data were collected anonymously through the online survey platform and stored securely in accordance with applicable data protection regulations. Only the research team had access to the data. The results are reported in aggregate form to prevent identification of individual participants. No financial or other incentives were provided for participation.

## Results

### Sample Characteristics

The study website received 501 unique visitors, of whom 122 provided informed consent. Among these, 111 respondents started the survey, and 81 (73%) completed it. Only completers were included in the analysis. Sample characteristics of the respondents can be found in Table 1. The most common free-text answer in the “Other” category for “Level of care” was working in a private practice (n=3), while 10 respondents did not provide an explanation.

**Table 1:** Sample characteristics of the respondents (N=81). Percentages may not total 100% due to rounding.

Characteristics		Values, n (%)
**Age (years)**		
	25-34	23 (28)
	35-44	30 (37)
	45-54	15 (18)
	55-64	10 (12)
	≥65	3 (4)
**Gender**		
	Women	58 (72)
	Men	19 (23)
	Nonbinary	1 (1)
	Unsure	1 (1)
	Prefer not to answer	2 (2)
**Organization**		
	Health care region	40 (49)
	Municipality	3 (4)
	Government	7 (9)
	Private	19 (23)
	Sole proprietorship	1 (1)
	Nonprofit or NGO^a^	8 (10)
	Other	3 (4)
**Level of care**		
	Primary care	21 (26)
	Specialized care	32 (40)
	Other	19 (23)
	Not applicable	9 (11)
**Targeted age group (years)**		
	Children (0-15)	14 (17)
	Adolescents or young adults (16-25)	10 (12)
	Adults (26-64)	56 (69)
	Older adults (≥65)	1 (1)
**Manager position**		
	Yes	7 (9)
	No	74 (91)

^a^NGO: nongovernmental organization.

### Experience of Refugee and Migrant Mental Health Interventions

Of respondents, 28 out of 81 (35%) reported that they worked in a setting that provided interventions for mental health problems that were specifically targeting refugees or migrants. A large proportion of respondents, 60 out of 81 (74%), reported having experience working with Arabic-, Dari-, or Farsi-speaking refugees or migrants. Most respondents did not speak Arabic, Dari, or Farsi, apart from 3 who reported being fluent in Dari or Farsi and 2 who had a limited understanding of Arabic. In terms of first-hand personal experience working with mental health problems in refugees, respondents stated that they had experience of psychotherapeutic or psychological treatments (48/81, 59%), assessment or diagnosis (41/81, 51%), psychosocial support (30/81, 37%), referrals (20/81, 25%), prevention (8/81, 10%), and medical or pharmacological treatment (2/81, 2%). A total of 15 respondents (18%) had no firsthand experience working with refugees or migrants.

### Use of Digital Solutions for Migrants and Refugees

Respondents reported limited availability of digital solutions at their workplace for addressing mental health problems among refugees and migrants (15/81, 19%). A large proportion of the sample responded that they were not using digital solutions for these groups, or they were using them only for other target groups (see Table 2).

**Table 2:** Psychologists reported use of digital assessment and treatment for refugees and migrants with mental health problems (N=81).

Reported use of solutions for refugees and migrants	Values, n (%)
Assessment or screening, and treatment	11 (14)
Assessment or screening	3 (4)
Treatment	2 (2)
No, but for other target groups	27 (33)
No use of digital tools	38 (47)

### Knowledge

The knowledge of digital interventions for refugees was limited in the sample, with 75% (61/81) rating their knowledge of digital screening and assessment as low or very low, and 72% (58/81) rating their knowledge of digital treatment formats similarly (see Table 3). More than half of the respondents (42/81, 52%) stated a lack of knowledge about the organization of care for refugees and migrants.

Explorative *t* tests indicated that those working in settings that provided interventions for refugees or migrants had significantly more knowledge of mental health assessment and treatment for refugees and migrants (*t*_79_=3.920; *P*<.001), guidelines and organization of care (*t*_79_=4.371; *P*<.001), and cultural considerations and adaptations (*t*_79_=2.269; *P*=.03). Similar results were found for those with personal experience of working with refugees or migrants (*t*_79_=3.446; *P*<.001; *t*_79_=3.079; *P*=.003; *t*_79_=2.925; *P*=.004) and those with specific experience of working with Dari-, Farsi-, and Arabic-speaking refugees or migrants (*t*_79_=3.892; *P*<.001; *t*_79_=2.189; *P*=.03; *t*_79_=2.722; *P*=.008). Respondents in workplace settings that provided interventions for refugees or migrants also had significantly higher knowledge of digital treatment formats (*t*_79_=2.978; *P*=.005). Those working in settings that provided digital formats for mental health problems for refugees or migrants and were themselves using digital formats for refugees or migrants had significantly higher scores on all 5 knowledge questions (*t*_79_=2.699 to 3.717; *P*<.001 to *P*=.01). Additionally, respondents employed at a health care region rated their knowledge of guidelines and organization of care for refugees significantly lower (*t*_79_=–2.419; *P*=.02), and the sensitivity analysis indicated that psychologists working in specialist care settings reported greater knowledge of digital screening and assessment tools (*U*=228; Z=–2.113; *P*=.04).

**Table 3:** Knowledge about the provision, organization, and cultural adaptation of mental health interventions for refugees and migrants (N=81).

Knowledge about	Frequency^a^	Mean (SD)
Assessment and/or treatment	1=6; 2=20; 3=30; 4=17; 5=8	3.08 (1.08)
Guidelines and organization of care for assessment and/or treatment	1=24; 2=18; 3=24; 4=8; 5=7	2.46 (1.26)
Digital assessment or screening tools	1=41; 2=20; 3=13; 4=4; 5=3	1.86 (1.09)
Digital treatment	1=33; 2=25; 3=13; 4=7; 5=3	2.04 (1.12)
Cultural adaptations of assessment and/or treatment	1=8; 2=21; 3=29; 4=14; 5=9	2.94 (1.13)

^a^Responses were measured on a 5-point Likert scale ranging from 1 (very low) to 5 (very high).

### Acceptability and Cultural Adaptation

Most respondents indicated a need to provide assessment and treatment in a digital format for refugees and migrants within the Swedish health care system (56/81, 69%). One in four selected the “Do not know” alternative (21/81, 26%). A significant proportion of respondents stated that digital solutions should be provided in the mother tongue of refugees and migrants (70/81, 86%) and that digital assessment and treatment should be culturally adapted (56/81, 69%). Those currently using, or having personal experience of, digital formats for refugees or migrants had significantly more “No” answers on the question if digital interventions should be culturally adapted than those without experience (*P*=.48; residual 2.3).

The respondents considered it most acceptable to provide digital treatment for refugees and migrants in the form of guided internet-based cognitive behavioral therapy (ICBT), by video, or in a blended treatment format (see Table 4). Respondents in specialist settings were significantly more positive about the use of video for refugees and migrants (*P*=.007) than those working in primary care, and women were more positive than men about self-help apps (*P*=.004).

**Table 4:** In what format can digital treatment be provided for refugees and/or migrants (multiple choice; N=81).

Digital format	Yes, n (%)	No, n (%)
Guided ICBT^a^	66 (82)	15 (18)
Unguided ICBT	42 (52)	39 (48)
Blended treatment	62 (77)	19 (23)
Internet-based or blended (other than CBT^b^)	47 (58)	34 (42)
Video	62 (77)	19 (23)
Self-help Apps	45 (56)	36 (44)
Chat	36 (44)	45 (56)
No digital treatment is suitable	2 (3)	79 (97)

^a^ICBT: internet-based cognitive behavioral therapy.

^b^CBT: cognitive behavioral therapy.

For most diagnoses and common mental health problems in refugees and migrants, a moderate level of severity was considered the highest level that could be treated using digital interventions (see Table 5). Fisher exact test showed that participants employed outside of a public health care region were more likely to report that moderate and severe depression could be treated with digital formats (*P*=.02). In the residual analysis, the “Moderate” response contributed 1.9, and the “Severe” response contributed 2.5. Respondents from public health care regions were more likely to report that posttraumatic stress disorder (PTSD) treatment for refugees and migrants was not suitable for digital formats (*P*=.04), with the “Not at all” response contributing 2.8 in the residual analysis. Those working in settings that had no digital formats for refugee or migrant mental health problems considered mild PTSD to be a more suitable option for digital treatment (*P*=.02).

**Table 5:** Highest level of severity that can be treated with digital interventions (N=81). Percentages may not total 100% due to rounding.

Diagnose	Not at all, n (%)	Mild, n (%)	Moderate, n (%)	Severe, n (%)	Do not know, n (%)
Depression	2 (2)	16 (20)	44 (54)	6 (7)	13 (16)
Social anxiety disorder	2 (2)	12 (15)	36 (44)	17 (21)	14 (17)
Panic disorder	1 (1)	17 (21)	33 (41)	15 (18)	15 (18)
Generalized anxiety disorder	7 (9)	21 (26)	28 (35)	11 (14)	14 (17)
PTSD^a^	7 (9)	21 (26)	28 (35)	11 (14)	14 (17)
Insomnia	2 (2)	10 (12)	31 (38)	24 (30)	14 (17)
Complicated grief	3 (4)	17 (21)	25 (31)	15 (18)	21 (26)

^a^PTSD: posttraumatic stress disorder.

### Preferred Provision of Care and Access to Digital Tools

Most respondents answered “No” to the question of whether refugees and migrants receive the care they need through Sweden’s health care system (58/81, 72%). Only 4 said “Yes” (5%), and 19 (23%) chose to answer, “Do not know.” Respondents in workplace settings that provided interventions for refugees or migrants had a higher proportion indicating that refugees and migrants do not receive the mental health care they need in the current health care system (*P*=.04; residual=2.6). Those in settings that did not provide such interventions had a higher proportion of “Do not know” responses (*P*=.04; residual=2.0).

Eighteen (22%) respondents worked in settings where digital treatment was already provided, and the other respondents were divided on whether such interventions should be implemented at their workplace (31/81, 38%) or not (32/81, 40%). Those working in settings that did not provide digital formats for refugee or migrant mental health had a significantly higher number of “Does not, but should” responses than those working in settings that did (*P*<.001; residual=2.2). Respondents preferred interventions for refugees and migrants to be provided through specialized units in primary care (38/81, 47%), followed by decentralized dissemination in primary care (28/81, 35%) and secondary or specialized care (6/81, 7%). Significantly more respondents working at the primary care level at the time of this writing believed that digital formats should be provided through specialist settings than those already working in such settings (*P*=.03; residual 2.6). In contrast, respondents working in specialist care settings were more likely to believe that dissemination should occur in nonspecialized settings in primary care compared to psychologists working there (*P*=.03; residual 1.9).

## Discussion

### Principal Findings

In this survey, 81 psychologists working primarily in public, private, and NGO health care settings provided their perspectives on the use of digital formats for the assessment and treatment of common mental health problems in refugees and migrants. Results from the survey showed that guided ICBT, blended treatment, and videoconferencing therapy were the most accepted digital formats for treating refugee and migrant mental health problems, although respondents had limited experience and low knowledge of using these or other digital solutions for refugees and migrants. There was a strong consensus in favor of providing digital solutions in refugees’ native language. Although a majority believed that interventions should also be culturally adapted for better fit, explorative analyses showed that those currently working with digital formats for refugees or migrants considered cultural adaptations to be less necessary. Psychologists preferred digital solutions to be delivered at the primary care level, either through centralized units or a decentralized approach.

### Role of Knowledge and Personal Experience

The lack of knowledge and use of digital formats for refugees and migrants among respondents highlights the current landscape in digital mental health, where interventions and assessment procedures are mainly targeting (and being created for) Western, native-speaking populations [56]. This lack of personal experience and knowledge among psychologists could act as a barrier to the use of digital formats, as seen in other studies [57].

Since increasing knowledge about digital solutions has been shown to increase perceived advantages and reduce perceived disadvantages of digital mental health interventions [58], awareness- and knowledge-raising activities for psychologists could serve as an important first step in health care settings where implementation of digital formats is being discussed or planned. The use of digital formats among psychologists in Sweden may facilitate the transition to similar alternatives for refugees and migrants, as therapists’ experience in delivering online mental health interventions during COVID-19 was associated with higher acceptance of these formats [59]. For those not using digital formats at the time of this writing, there is a need to examine how such a transition is perceived [44], recognizing that those transitioning may perceive both negative and mixed views on using new technologies for the provision of care [60].

### Positive Attitudes and Preferred Digital Solutions

The preference for ICBT, videoconferencing, and blended treatment among respondents may not be surprising, as these are the most commonly used digital delivery formats in the Swedish health care system by psychologists and have been widely studied by researchers [19,27,61]. An interesting finding was that guided ICBT received more positive ratings than blended treatment, which contrasts with earlier surveys showing the opposite [26,57,62]. This could be due to the fact that ICBT programs have been available for 8 years through the public national online platform for internet-delivered treatment and support [63] and are frequently delivered by psychologists. Additionally, it may be preferred because it can facilitate the provision of evidence-based treatment in Arabic, Dari, or Farsi, without requiring the psychologist to be fluent in these languages, thus overcoming the barrier of using an interpreter [64]. The positive attitudes toward the use of video in specialist settings may be explained by the fact that it was the preferred digital solution in the transition to digital formats in psychiatric care during the COVID-19 pandemic [45]. Another perspective is that videoconferencing therapy has been used for diagnoses often treated in specialist settings (eg, PTSD) and can be perceived as more easily adapted for more severe conditions than other digital formats [21]. This and the generally positive attitudes toward digital interventions expressed by psychologists, including the use of smartphone apps among women, could facilitate the transition to digital alternatives, as acceptability has an impact on implementation and scalability [65].

### Need for Translated and Culturally Adapted Solutions

Even though most respondents had at least some experience working with refugees or migrants, including working with Arabic-, Dari-, or Farsi-speaking patients, no psychologists in this survey could speak Arabic fluently. Since Arabic is the second most widely spoken mother tongue in Sweden [49], and a significant proportion of refugees originate in countries where Arabic, Dari, or Farsi is spoken [48], there is an obvious gap between the need for linguistically adapted solutions and the language skills of psychologists providing interventions. Increasing the workforce by employing psychologists who speak Arabic, Dari, or Farsi as their mother tongue or educating culture brokers to facilitate understanding between patients and health care providers [10] are viable options but will take time and not be sufficient. Instead, using translated assessment and treatment materials or facilitating the use of interpreters in digital formats, such as video, could swiftly increase access by adapting existing technology and content. The preference for culturally adapted digital solutions seen in this study has been confirmed by other studies, for example, in a survey of New Zealand mental health clinicians, where three-quarters expressed a need to tailor solutions for specific populations, including immigrants and refugees [62]. As existing frameworks simplify the process of cultural adaptation [35,66,67], and culturally adapted interventions have been found to lead to better outcomes [37], adapting digital interventions could increase the possibility of dissemination in routine care settings. An interesting finding in our exploratory analysis was that those already working with digital interventions for refugees or migrants considered cultural adaptations less necessary. It could be that these psychologists had prior experience with nonadapted versions being sufficient, which led them to answer “No” to the question of whether cultural adaptations were necessary. This finding can be seen in the light of a recent meta-analysis that found no added effects for culturally adapted digital interventions in low-income settings [68], making the question of cultural adaptations a complex issue that requires further examination [69].

### Appropriate Level of Severity for Digital Interventions

Given that there are few studies on digital interventions specifically targeting refugees [70] and migrants [71,72] at the time of writing, the acceptability among respondents of digital interventions for conditions such as depression and panic disorder could be influenced by research on internet-delivered interventions in general, which supports the efficacy for mild to moderate common mental health problems [73]. An increase in digital mental health research targeting refugees and migrants could help inform key stakeholders, making their opinions more based on evidence and less dependent on personal preferences and attitudes toward digital formats. The acceptability of using digital solutions for more severe mental health problems, such as for severe levels of insomnia, social anxiety, and depression in this study, confirms findings from other surveys [26,74]. This suggests that using digital interventions for more difficult psychiatric conditions should be examined further, both from an effectiveness and provider perspective. On the other hand, respondents working in a public health care region and those working in settings where digital solutions for refugees and migrants were not available responded more cautiously about using digital formats for PTSD. Since PTSD is more common among refugees [4] and often treated in specialized settings in Sweden, the lower degree of acceptability could be attributed to it being considered a more difficult diagnosis to treat, contributing to it being viewed as unsuitable for digital formats or only preferable for milder forms of mental health problems. New innovative digital interventions for PTSD could also have the potential to increase acceptability, being more adapted to refugee needs and degree of complexity [75].

### Provision of Care and the Perceived Treatment Gap

The already recognized gap in both high- and low-income settings between the mental health burden of refugees and migrants and their access to care [76,77] was confirmed by respondents in this survey. If digital interventions are to help close this gap, it is essential to understand how they should be provided in routine care settings [78], including through broad dissemination that extends beyond trauma-focused treatment in specialized settings [64]. An interesting finding was that respondents in specialized settings preferred implementation in primary care settings, and vice versa, which highlights the difficulty of organizing care for refugees in the existing health care systems. ICBT has been organized in both a centralized (specialized) and decentralized fashion in Sweden [15]. When comparing 2 organizational models in Swedish primary care, a decentralized and a more concentrated model, both models had specific advantages that could make them viable options also from a refugee perspective [79]. A centralized model could increase therapist and organizational competence of refugee mental health and provision of adapted digital interventions, but a decentralized model could make digital treatment more accessible to refugees as they receive their care in the same setting as others enduring mild to moderate mental health problems. When views of outpatient mental health care deliverers (psychotherapists and psychiatrists) were investigated in a large survey study in Switzerland, almost half of the respondents reported having treated refugees and asylum seekers within the last 12 months with a median waiting time of only 3 weeks [64]. This result indicates that a more decentralized model of provision, which could include digital interventions, has the possibility of increasing access to care for refugees compared to more selective and highly specialized services.

As implementation research highlights the importance of decisions and involvement in outer and inner contexts, along with the influence of individual stakeholders, in successful implementation efforts [80], it is crucial to ensure psychologists feel involved in the setup and implementation of digital solutions. Free text responses on potential gatekeepers showed that respondents were unsure of which individuals or organizations were the most important for the facilitation of successful implementation and stated that gatekeepers were politicians and leaders in health care settings, not health care staff. This finding, along with other data from this study showing generally low knowledge about the organization of care for refugees, may also highlight a knowledge and perceived influence gap, not only in the provision of care but also in understanding the organization of care and the role of one’s own profession. One way forward could therefore be to increase the interest of professional psychology societies in helping to establish clinical guidelines and influence the organization and provision of digital mental health in health care contexts [81].

### Limitations

The sample size was very small for a national survey and restricts the generalizability of the findings and increases the risk of overinterpretation. As such, all findings should be interpreted cautiously. Also, the use of convenience sampling may introduce self-selection bias into the sample and does not permit an accurate estimation of the number of potential respondents who received information about the survey. To contextualize the sample characteristics, we compared the demographic distribution of respondents with national data on licensed psychologists in Sweden. The sample showed a similar distribution with respect to age and gender [82,83]. However, due to the convenience-based recruitment strategy, the findings cannot be considered representative of all psychologists in Sweden. The survey may also have attracted individuals with an existing interest in digital solutions, and results could reflect a more positive view of digital interventions than what is typical among psychologists. Although the survey aimed to include clinical psychologists in health care settings, 9 (11%) respondents reported that the question about level of care (eg, primary or specialized) was not applicable to their work. These respondents may therefore not fully represent a health care context, which should be considered when interpreting the results.

There are several commonly used terms that overlap in their definition and are closely related, such as “migrant,” “immigrant,” “refugee,” “asylum seeker,” and “forcibly displaced individuals.” The term “asylum seeker,” as well as distinctions within refugees and migrants based on residence status (no permit, temporary permit, and permanent permit), was not included in the survey. This omission may have reduced internal validity, as respondents might have answered differently had the survey differentiated between individuals in the asylum process and those with temporary or permanent residence permits, given the legal differences in access to health care and exposure to mental health stressors across these groups. Our definitions of refugees, migrants, and digital solutions were included in the introduction to the survey (see Multimedia Appendix 7) and were based on established terminology and a consensus process within the research group. We received no feedback from the participants indicating that further clarification was needed regarding the terms used in the survey.

The inferential analyses were exploratory in nature, as no a priori power analysis was conducted, and the sample size was relatively small. To facilitate statistical analysis and increase the number of data points in each cell, survey responses were combined into binary categories. This categorization may limit the conclusions drawn from the inferential statistics used in the study. The survey included 3 open-ended questions on the used digital tools, preferred digital tools, and gatekeepers for dissemination. Responses were not subjected to formal content analysis procedures due to the design of the questions and the nature of the material provided by participants, which primarily consisted of short answers or simple descriptions.

### Future Directions

Psychologists can strongly influence the provision of care and the implementation of new methods, including digital mental health interventions. It is therefore important to examine further how they and other mental health professionals perceive how and if digital interventions for refugees and migrants should be implemented in a health care context [42]. Understanding the perspectives of psychologists who have yet to use digital formats is vital to address potential resistance and ensure successful implementation. There is a need for further stakeholder surveys, in-depth qualitative interviews, continuous monitoring of psychologists’ knowledge and skills, and increased use of specific implementation frameworks to guide further analysis and dissemination efforts. Strategies to enhance learning and acceptance of digital formats among health care professionals are crucial, as awareness-raising activities and targeted training programs could help reduce barriers and lead to greater adoption and inclusion of marginalized groups such as refugees and migrants.

### Conclusions

This survey represents an important first step in understanding psychologists’ perspectives on using digital mental health solutions for refugees and migrants in Sweden. While knowledge and experience were limited, attitudes were generally positive, with guided ICBT, blended treatment, and videoconferencing therapy being the most accepted formats. The emphasis on linguistic accessibility underscores the need for adapted solutions in mental health care provision for refugees and migrants. However, the need for cultural adaptation is more complex and requires further exploration. Despite limitations, such as potential self-selection bias and exploratory statistical analyses, the findings provide valuable insights that can inform future research and implementation efforts. Addressing knowledge gaps, fostering acceptance of digital solutions, and developing scalable, adapted interventions are crucial steps toward alleviating the mental health burden for refugees and migrants in Sweden and beyond.

## Appendix

Multimedia Appendix 1 Filled out version of the Checklist for Reporting Results of Internet E-Surveys (CHERRIES).

Multimedia Appendix 2 English translation of the recruitment material used to invite participants to the online survey.

Multimedia Appendix 3 English translation of full online survey.

Multimedia Appendix 4 Description of the Iterapi platform used for survey administration and data collection.

Multimedia Appendix 5 Full list of recategorized variables used for statistical analyses.

Multimedia Appendix 6 English translation of digital informed consent form presented to participants prior to accessing the online survey.

Multimedia Appendix 7 Definitions of specific terms used in the survey.

## Abbreviations

CHERRIES: Checklist for Reporting Results of Internet E-Surveys
ICBT: internet-based cognitive behavioral therapy
NGO: nongovernmental organization
PTSD: posttraumatic stress disorder

## Data Availability

The datasets generated or analyzed during this study are available from the corresponding author for noncommercial purposes on reasonable request.
