# Supplementary material for: Understanding Psychologists’ Usage, Knowledge, and Attitudes Toward Digital Mental Health Solutions for Refugees and Migrants: Exploratory Cross-Sectional Survey in Sweden
Source: JMIR Hum Factors. 2026 Mar 3;13:e75263. doi: 10.2196/75263 (PMC12996901; doi:10.2196/75263)
Supplement: Multimedia Appendix 1 [file humanfactors_v13i1e75263_app1.docx]

# Multimedia Appendix 1

# Checklist for Reporting Results of Internet E-Surveys (CHERRIES)

| ***Checklist Item*** | ***Explanation*** |
| --- | --- |
| Describe survey design | Convenience sampling was used to collect the data. An online self-report survey was distributed between December 2023, and February 2024. Survey respondents were licensed psychologists working mainly in private, public, or non-governmental settings in Sweden. |
| IRB approval | Ethical approval was obtained from the Swedish Ethical Review Authority (Approval ID: 2022-04274-02). |
| Informed consent | Posts and emails described the study as a survey for psychologists about their perspectives on using digital solutions for mental health problems in refugees and migrants and included a link to the online survey. Respondents were informed that the questionnaire was estimated to take about 15-20 minutes, that participation was anonymous. and that no prior knowledge of the survey subject was required to participate in the survey. Invitations included a brief description of the study, the intended respondents, and a link to the survey. Participation was voluntary and participants were provided informed consent through the online survey platform and had to consent digitally before answering the questionnaire. Respondents could not access the survey if declining informed consent.  Respondents were informed that information provided in the survey will be kept in an encrypted database and stored for at least 10 years after completion of the study and that answers will be treated confidentially and in anonymized form. Information was provided that the survey was part of the SAHA-project and that the survey was conducted by researchers at Linköping University, Mid Sweden University, Stockholm University, and Karolinska Institutet. |

|  |  |
| --- | --- |
| Data protection | Excerpt from Multimedia Appendix 4:    The Iterapi platform used for this survey is a secure and widely used solution for providing online surveys, questionnaires, and internet-based interventions. The servers are physically located at Linköping University, Sweden, in a locked computer room accessible only to authorized personnel. A responsive website compatible with web browsers on mobile phones, tablets, and computers was set up to provide information about the study, including the possibility to register and provide informed consent. All communication between servers and users was encrypted, and the survey utilized its own dedicated database, configuration files, database passwords, and encryption keys. Survey data was stored in an encrypted format within the database, using advanced algorithms and secret keys to prevent unauthorized linkage between stored data and individual users. The servers, where the data was stored, were continuously monitored and updated following well-defined routines by the local security team at Linköping University. Access to user data was restricted to authorized members of the research team. The website and survey were open-access, meaning they were not password-protected and could be accessed by anyone with the survey link. To maintain anonymity, no client IP addresses were collected or stored. A cookie was used to prevent duplicate responses from the same browser and to recall partially saved answers if respondents completed the survey in multiple sessions. Respondents were able to revise their answers while completing the survey by using the Back and Forward buttons. Once the survey was submitted, answers could no longer be modified. There was no function for the participants to download their survey answers during or after the completion of the survey. |
| Development and testing | The survey was developed by researchers at Linköping University and Karolinska Institutet. The first draft was reviewed by researchers with expertise in digital assessment and treatment, refugee and migrant populations, and cultural adaptation. Revisions addressed terminology, clarity, thematic coverage, and alignment with analytic constructs. A beta version was further tested by the research group and psychologists from public, private, and NGO sectors to ensure contextual fit, leading to additional refinements before finalizing the survey. Key terms, including migrant and refugee, were defined and used consistently throughout.  The survey was administered in Swedish via the Iterapi platform, a secure and extensively used platform for collecting research data online, including questionnaires and surveys. The user experience of the online survey was tested continuously by the webmaster and main author whenever revisions were made to content of the survey. The length of the survey was taken into consideration during the development to ensure that the survey required approximately 15-20 minutes to complete. Closed items included binary, nominal, multiple-choice, and five-point Likert scale formats. |

| Open survey versus closed survey | The website and survey were open-access, meaning they were not password-protected and could be accessed by anyone with the survey link. To maintain anonymity, no client IP addresses were collected or stored. A cookie was used to prevent duplicate responses from the same browser and to recall partially saved answers if respondents completed the survey in multiple sessions. |
| --- | --- |
| Contact mode | The study information and invitation to participate in the survey was distributed using a variety of digital channels. Emails with study information were sent to selected representatives for distribution within their organizations. Information was also distributed through various mailing lists and social media posts, including in several professional Facebook Meta groups for psychologists (≈200-10000 members). Data on actual reach through these different distribution channels are not available. The information provided described the study as a survey for psychologists about their perspectives on using digital solutions for mental health problems in refugees and migrants. It also included a link to the online survey. Reminders were sent once or twice through these distribution channels. |
| Advertising the survey | Advertisement of the survey was done through online groups, mailing lists, and selected representatives (see contact mode). No paid online marketing channels were used. The material used to describe the survey can be found in Multimedia Appendix 2. |
| Web/E-mail | The survey was web-based using a responsive website (with a dedicated URL) compatible with web browsers on mobile phones, tablets, and computers was set up to provide information about the study, including the possibility to register and provide informed consent. |

| Context | The website was created in Iterapi for the online survey with a dedicated URL not used for anything else than the survey. The link to the survey was provided in the information material. |
| --- | --- |
| Mandatory/voluntary | Participation in the online survey was voluntary. Respondents had to provide informed consent before starting the survey. |
| Incentives | No monetary incentives were offered, and respondents consented to provide their responses voluntarily. |

| Time/Date | Data collection took place between December 2023 and February 2024. |
| --- | --- |
| Randomization of items or questionnaires | Items were not randomized. |
| Adaptive questioning | The online questionnaire included one adaptive question. If respondents chose answer 5 on item 13 (“No, I do not currently use digital formats for either assessment or treatment of mental health problems”), they were not provided item 14 (“If you are using digital assessment tools, online questionnaires, internet-based treatment/ICBT-programs, digital support programs, video sessions, apps, or other digital formats at your workplace, please provide specific examples of which of these you are using”) |
| Number of Items | The online survey included 26 closed questions, five open ended free text questions, and one optional free text “Other comments” as the final item |
| Number of screens (pages) | The survey consisted of nine webpages, including informed consent and a final page with repeated key information about the study. Questions were grouped based by theme, with each page containing between one and eight questions. |

|  |  |
| --- | --- |
| Completeness check | All items in the survey, except item 32, were mandatory and selection of at least one response option was always enforced. At the end of the survey, respondents submitted the survey by clicking on the “submit” button. A submit date with a time stamp was saved in the platform. |
| Review step | Respondents were able to revise their answers while completing the survey by using the Back and Forward buttons. Once the survey was submitted, answers could no longer be modified. |
| Unique site visitor | To maintain anonymity, no client IP addresses were collected or stored. A cookie was used to prevent duplicate responses from the same browser and to recall partially saved answers if respondents completed the survey in multiple sessions. |
| View rate (Ratio of unique survey | The study website received 501 unique visitors. 122 provided informed consent and 111 started the survey. View rate was 22% (111/501). |

| visitors/unique site visitors) |  |
| --- | --- |
| Participation rate (Ratio of unique visitors who agreed to participate/unique first survey page visitors) | 122 provided informed consent and 111 started the survey. The participation rate was 91% (111/122). |
| Completion rate (Ratio of users who finished the survey/users who agreed to participate) | 111 respondents started the survey, and 81 completed it. The completion rate was 73% (81/111). |
| Cookies used | A cookie was used to prevent duplicate responses from the same browser and to recall partially saved answers if respondents completed the survey in multiple sessions. |

| IP check | To maintain anonymity, no client IP addresses were collected or stored. |
| --- | --- |
| Log file analysis | No other techniques were used for identification of multiple entries. |
| Registration | The survey was open-access and did not require log in or registration. |
| Handling of incomplete  questionnaires | Only complete survey responses were analyzed. |

|  |  |
| --- | --- |
| Questionnaires submitted with an atypical timestamp | Timestamps were not used as a criterion. |
| Statistical correction | No such methods were used. Responses from certain items were recoded into binary variable to ensure adequate cell sizes in correlational analyses and t-test (see Multimedia Appendix 5). |

This checklist has been modified from Eysenbach G. Improving the quality of Web surveys: the Checklist for Reporting Results of Internet E-Surveys (CHERRIES). J Med Internet Res. 2004 Sep 29;6(3):e34 [erratum in J Med Internet Res. 2012; 14(1): e8.]. Article available at [https://www.jmir.org/2004/3/e34/;](https://www.jmir.org/2004/3/e34/%3B) erratum available [https://www.jmir.org/2012/1/e8/.](https://www.jmir.org/2012/1/e8/) Copyright ©Gunther Eysenbach. Originally published in the Journal of Medical Internet Research, 29.9.2004 and 04.01.2012.

This is an open-access article distributed under the terms of the Creative Commons Attribution License ([https://creativecommons.org/licenses/by/2.0/),](https://creativecommons.org/licenses/by/2.0/)) which permits unrestricted use, distribution, and reproduction in any medium, provided the original work, first published in the Journal of Medical Internet Research, is properly cited.
