# Supplementary material for: Understanding Psychologists’ Usage, Knowledge, and Attitudes Toward Digital Mental Health Solutions for Refugees and Migrants: Exploratory Cross-Sectional Survey in Sweden
Source: JMIR Hum Factors. 2026 Mar 3;13:e75263. doi: 10.2196/75263 (PMC12996901; doi:10.2196/75263)
Supplement: Multimedia Appendix 2 [file humanfactors_v13i1e75263_app2.docx]

**Multimedia Appendix 2 – Recruitment material**

The version below was translated with DeepL Pro on January 9, 2025. For the original Swedish version, please contact the authors.

Survey for psychologists! 👇

[www.iterapi.se/enkat/saha](http://www.iterapi.se/enkat/saha)

We are currently collecting data for a multi-year research project on digital interventions for mental health problems in refugees and migrants where we want to know more about psychologists' perspectives and use of digital formats in assessment and treatment. The project is a collaboration between Linköping University, Karolinska Institutet, Mid Sweden University and Stockholm University.

***You do not need to work with digital interventions for refugees or migrants to fill out the survey, we are interested in your answers regardless of your previous experience and knowledge.*** The survey will take about 15-20 minutes to complete and will contribute to increased knowledge about the conditions for implementing digital interventions for refugees and migrants in routine care.

Thank you for your time!

For questions about the study, please contact:

Kristofer Vernmark

Licensed psychologist and PhD at Linköping University

E-mail: [kristofer.vernmark@liu.se](mailto:kristofer.vernmark@liu.se)

Anahita Geranmayeh

PhD student at Karolinska Institutet

E-mail: anahita.geranmayeh@ki.se

Read more about the SAHA project here: <https://liu.se/forskning/saha>
