# Supplementary material for: Understanding Psychologists’ Usage, Knowledge, and Attitudes Toward Digital Mental Health Solutions for Refugees and Migrants: Exploratory Cross-Sectional Survey in Sweden
Source: JMIR Hum Factors. 2026 Mar 3;13:e75263. doi: 10.2196/75263 (PMC12996901; doi:10.2196/75263)
Supplement: Multimedia Appendix 3 [file humanfactors_v13i1e75263_app3.docx]

**Multimedia Appendix 3 – SAHA Stakeholder survey (SAHA-S)**

The version below was translated with the help of DeepL Pro on December 17, 2024 and minor changes were made to the translation on October 7, 2025, to increase clarity. Users of the survey are encouraged to acknowledge the authors of this article. For the original Swedish version of the survey, please contact the authors.

1. **What age are you?**
2. 18-24
3. 25-34
4. 35-44
5. 45-54
6. 55-64
7. 65+
8. **What is your gender? By gender we mean gender identity, i.e. the gender you identify yourself as.**
9. Female
10. Man
11. Non-binary
12. Other option
13. Uncertain
14. Prefer not to answer
15. **Who is your main employer?**
16. Healthcare region
17. Municipality
18. Government
19. Private company
20. Sole proprietorship
21. Non-profit (e.g. NGO)
22. Other: free text
23. **Do you work in any of the following levels of care (if you work in several levels of care, please select your primary level)?**
24. Primary care
25. Specialist care
26. Other: free text
27. Not applicable
28. **Do you hold a managerial position in your organization?**
29. Yes
30. No
31. **What age group do you mainly work with?**
32. Children (0-15)
33. Adolescents/Young adults (16-25)
34. Adults (26-64)
35. Older adults (65+)
36. **In your workplace, are there interventions specifically targeting the mental health/illness of refugees and/or migrants?**
37. Yes
38. No
39. **Do you have personal experience working with refugees and/or migrants with mental health problems (multiple choice)?**

- Yes, psychological/psychotherapeutic treatment (Yes/No)
- Yes, medical/pharmacological treatment (Yes/No)
- Yes, psychosocial support (e.g. counseling and support sessions) (Yes/No)
- Yes, assessment/diagnosis (Yes/No)
- Yes, prevention (Yes/No)
- Yes, referral internally or to another caregiver (Yes/No)
- No, I do not work with refugees and/or migrants' mental health (Yes/No)
- Other: free text

1. **Do you have personal experience working with refugees and/or migrants whose mother tongue is Persian, Dari, Farsi or Arabic?**
2. Yes, Arabic and Persian, Dari or Farsi
3. Yes, Persian, Dari or Farsi
4. Yes, Arabic
5. No
6. **Do you speak Arabic?**
7. Mother tongue
8. Fluent in speech and writing (not native speaker)
9. Limited understanding
10. No
11. **Do you speak Persian, Dari or Farsi?**
12. Mother tongue
13. Fluent in speech and writing (not native speaker)
14. Limited understanding
15. No
16. **At your workplace, do you use any digital solutions specifically targeting mental health/illness in refugees and/or migrants?**
17. Yes
18. No
19. **Do you currently use digital formats in your work with refugees' and/or migrants' mental health/illness?**
20. Yes, both in assessment and treatment
21. Yes, only for assessment
22. Yes, only for treatment
23. No, but I use digital formats for mental health assessment and/or treatment for target groups other than refugees/migrants
24. No, I do not currently use digital formats for either assessment or treatment of mental health problems
25. **If you are using digital assessment tools, online questionnaires, internet-based treatment/ICBT-programs, digital support programs, video sessions, apps, or other digital formats at your workplace, please provide specific examples of which of these you are using**- Free text
26. **How would you rate your current level of knowledge regarding the assessment and/or treatment of refugees' and/or migrants' mental health problems?**

Very low - Very high (1-5)

1. **How would you rate your current level of knowledge regarding guidelines and organization of care for the assessment and/or treatment of refugees and/or migrants with mental health problems?**

Very low - Very high (1-5)

1. **How would you rate your current level of knowledge about digital screening tools and assessment forms for mental health problems in refugees and/or migrants?**

Very low - Very high (1-5)

1. **How would you rate your current level of knowledge about digital treatment formats (e.g. internet-based CBT, videoconferencing therapy, VR-treatment) for mental health problems in refugees and/or migrants?**

Very low- Very high (1-5)

1. **How would you rate your current level of knowledge about cultural considerations and adaptations in the assessment and/or treatment of mental health problems for refugees and/or migrants?**

Very low- Very high (1-5)

1. **Do you think there is a need to offer digital mental health interventions for refugees and/or migrants in Swedish healthcare (multiple choice)?**
2. Yes, to assess and treat mental health problems
3. Yes, to assess mental health problems
4. Yes, to treat mental health problems
5. No
6. Do not know
7. **Do you think that refugees and/or migrants should have access to digital mental health interventions in their native language in Swedish healthcare?**
8. Yes
9. No
10. Do not know
11. **Do you think that digital solutions to assess and treat refugees should be specifically adapted to the target group's norms, values and views on mental health, so-called culturally adapted interventions?**
12. Yes
13. No
14. Do not know
15. **Do you think it is appropriate to provide digital treatment for mental health problems in refugees and/or migrants in Swedish healthcare in any of the following ways (multiple choice)?**

- Via Internet-delivered CBT programs (ICBT) **with** therapist support (Yes/No)
- Via Internet-delivered CBT programs (ICBT) **without** therapist support (Yes/No)
- Via blended treatment (internet-based CBT material + sessions with therapist) (Yes/No)
- Via internet-delivered programs or blended treatment with other orientation than CBT (Yes/No)
- Via videoconferencing (Yes/No)
- Via self-help apps (Yes/No)
- Via Chat (Yes/No)
- Refugees and/or migrants should not be offered digital mental health interventions (Yes/No)
- Other (free text)

1. **What is the most severe level of mental illness in refugees and/or migrants that you think can be treated with psychological/psychotherapeutic interventions in a digital format for the following problems?**

Depression (1=not at all, 2=mild, 3=moderate, 4=severe, 5=do not know)

Social anxiety (1=not at all, 2=mild, 3=moderate, 4=severe, 5=do not know)

Panic disorder (1=not at all, 2=mild, 3=moderate, 4=severe, 5=do not know)

Generalized anxiety disorder (1=not at all, 2=mild, 3=moderate, 4=severe, 5=do not know)

PTSD (1=not at all, 2=mild, 3=moderate, 4=severe, 5=do not know)

Insomnia (1=not at all, 2=mild, 3=moderate, 4=severe, 5=do not know)

Complicated grief (1=not at all, 2=mild, 3=moderate, 4=severe, 5=do not know)

1. **Which of the following statements do you agree with the most:**
2. Refugees and/or migrants should be offered treatment for mild to moderate mental health problems in **primary care**, just like other target groups with the same level of problems
3. Refugees and/or migrants should be offered treatment for mild to moderate mental problems in **specialized units at the primary care level** where there is more expertise and resources to address the target group
4. Refugees and/or migrants should be offered treatment for mild to moderate mental health problems at a **specialist level** as there is a higher degree of complexity in the target group's problems that requires specialist expertise to treat
5. I disagree with any of the above and believe that treatment interventions for refugees and/or migrants should be organized differently (please provide answers in free text)
6. **From your perspective, do refugees and/or migrants in Sweden today receive the mental health care they need in Swedish healthcare system?**
7. Yes
8. No
9. Do not know
10. **Do you think your organization/workplace should offer digital treatment formats for refugees and/or migrants with mental health problems?**
11. Yes, it already does
12. Yes, it does not but it should
13. No, it should not

We would like you to answer the following four final questions in free text. Your views are important in order to provide the best possible basis for the survey, so we appreciate you taking the time to think about and elaborate on your answers to these questions.

1. **What kind of *digital tools* would you have liked your organization/workplace to have access to in the assessment and treatment of refugees and/or migrants' mental health? (If you do not want any digital tools, you can leave this box blank)**

- Free text

1. **What are the main *barriers/challenges* to introducing and disseminating digital formats for the assessment and treatment of mental health problems in refugees and/or migrants in Swedish healthcare (in your or other organization)?**

- Free text

1. **What are the main *enabling/facilitating* factors for introducing and disseminating digital formats for the assessment and treatment of mental health problems in refugees and/or migrants in Swedish healthcare (in your or other organizations)?**

- Free text

1. **Which *actors (organizations and/or roles)* within the Swedish healthcare system do you think have the greatest impact on the introduction and dissemination of digital formats for mental health in refugees and/or migrants in Swedish healthcare?**

- Free text

1. **Other comments (optional)**

- Free text
