# Supplementary material for: Understanding Psychologists’ Usage, Knowledge, and Attitudes Toward Digital Mental Health Solutions for Refugees and Migrants: Exploratory Cross-Sectional Survey in Sweden
Source: JMIR Hum Factors. 2026 Mar 3;13:e75263. doi: 10.2196/75263 (PMC12996901; doi:10.2196/75263)
Supplement: Multimedia Appendix 4 [file humanfactors_v13i1e75263_app4.docx]

**Multimedia Appendix 4 – Description of the online survey platform (Iterapi)**

The Iterapi platform used for this survey is a secure and widely used solution for providing online surveys, questionnaires, and internet-based interventions [1]. The servers are physically located at Linköping University, Sweden, in a locked computer room accessible only to authorized personnel. A responsive website compatible with web browsers on mobile phones, tablets, and computers was set up to provide information about the study, including the possibility to register and provide informed consent. All communication between servers and users was encrypted, and the survey utilized its own dedicated database, configuration files, database passwords, and encryption keys. Survey data was stored in an encrypted format within the database, using advanced algorithms and secret keys to prevent unauthorized linkage between stored data and individual users. The servers, where the data was stored, were continuously monitored and updated following well-defined routines by the local security team at Linköping University. Access to user data was restricted to authorized members of the research team.

The website and survey were open-access, meaning they were not password-protected and could be accessed by anyone with the survey link. To maintain anonymity, no client IP addresses were collected or stored. A cookie was used to prevent duplicate responses from the same browser and to recall partially saved answers if respondents completed the survey in multiple sessions. Respondents were able to revise their answers while completing the survey by using the Back and Forward buttons. Once the survey was submitted, answers could no longer be modified. There was no function for the participants to download their survey answers during or after the completion of the survey.

The survey consisted of nine webpages, including informed consent and a final page with repeated key information about the study. Questions were grouped based by theme, with each page containing between one and eight questions.

No automatic filtering was applied. For those visiting the study webpage without checking the informed consent box (n=389), no other information than time and date of access was recorded. The survey could not be accessed without providing informed consent.

**References**

1. Vlaescu G, Alasjö A, Miloff A, Carlbring P, Andersson G. Features and functionality of the Iterapi platform for internet-based psychological treatment. *Internet Interventions*. 2016;6:107-114. doi:10.1016/j.invent.2016.09.006
