# Supplementary material for: Understanding Psychologists’ Usage, Knowledge, and Attitudes Toward Digital Mental Health Solutions for Refugees and Migrants: Exploratory Cross-Sectional Survey in Sweden
Source: JMIR Hum Factors. 2026 Mar 3;13:e75263. doi: 10.2196/75263 (PMC12996901; doi:10.2196/75263)
Supplement: Multimedia Appendix 5 [file humanfactors_v13i1e75263_app5.docx]

**Multimedia Appendix 5 – Recategorization of variables**

Categorization of data from questions 1 to 8, 12 and 13

1. **What age are you?**
2. 18-24*
3. 25-34
4. 35-44
5. 45-54
6. 55-64
7. 65+

2-3 = Category 1 (25-44), n=53

4-6 = Category 2 (45+ ), n=28

*There were no participants in the survey in this age group

1. **What is your gender? By gender we mean gender identity, i.e. the gender you identify yourself as.**
2. Female
3. Man
4. Non-binary
5. Other option
6. Uncertain
7. Prefer not to answer

1= Category 1 (Female), n=58

2= Category 2 (Male), n=19

3-6 = Excluded from analysis, n=4

1. **Who is your main employer?**
2. Healthcare region
3. Municipality
4. Government
5. Private company
6. Sole proprietorship
7. Non-profit (e.g. NGO)
8. Other: free text

1=Category 1 (Healthcare region), n=40

2-7= Category 2 (All others), n=41

1. **Do you work in any of the following levels of care (if you work in several levels of care, please select your primary level)?**
2. Primary care
3. Specialist care
4. Other: free text
5. Not applicable

1=Category 1 (Primary care), n=21

2=Category 2 (Specialist care), n=32

3-4 = Excluded from analysis, n=28

1. **Do you hold a managerial position in your organization?**
2. Yes
3. No

1=Category 1 (Manager), n=7

2=Category 2 (Not manager), n=74

1. **What age group do you mainly work with?**
2. Children (0-15)
3. Adolescents/Young adults (16-25)
4. Adults (26-64)
5. Older adults (65+)

1-2 = Category 1 (0-25, Children, Adolescents and Young adults), n=24

3-4 = Category 2 (26+, Adults and older adults), n=57

1. **In your workplace, are there interventions specifically targeting the mental health/illness of refugees and/or migrants?**
2. Yes
3. No

1=Category 1 (Refugee/migrant interventions at workplace), n=28

2=Category 2 (No refugee/migrant interventions at workplace), n=53

1. **Do you have personal experience working with refugees and/or migrants with mental health problems (multiple choice)?**

- Yes, psychological/psychotherapeutic treatment (Yes/No)
- Yes, medical/pharmacological treatment (Yes/No)
- Yes, psychosocial support (e.g. counseling and support sessions) (Yes/No)
- Yes, assessment/diagnosis (Yes/No)
- Yes, prevention (Yes/No)
- Yes, referral internally or to another caregiver (Yes/No)
- No, I do not work with refugees and/or migrants' mental health (Yes/No)
- Other: free text*

1-6 = Category 1 (Yes), n=66

7 = Category 2 (No), n=15

*There were no answers in this category

1. **Do you have personal experience working with refugees and/or migrants whose mother tongue is Persian, Dari, Farsi or Arabic?**
2. Yes, Arabic and Persian, Dari or Farsi
3. Yes, Persian, Dari or Farsi
4. Yes, Arabic
5. No

1-3 = Category 1 (Yes), n=60

1. = Category 2 (No), n=21

**12. At your workplace, do you use any digital solutions specifically targeting mental health/illness in refugees and/or migrants?**

1. Yes
2. No

1 = Category 1 (Yes), n=15

1. = Category 2 (No), n=66

**13. Do you currently use digital formats in your work with refugees' and/or migrants' mental health/illness?**

1. Yes, both in assessment and treatment
2. Yes, only for assessment
3. Yes, only for treatment
4. No, but I use digital formats for mental health assessment and/or treatment for target groups other than refugees/migrants
5. No, I do not currently use digital formats for either assessment or treatment of mental health problems

1-3 = Category 1 (Yes), n=16

4-5 = Category 2 (No), n=65
