# Supplementary material for: Understanding Psychologists’ Usage, Knowledge, and Attitudes Toward Digital Mental Health Solutions for Refugees and Migrants: Exploratory Cross-Sectional Survey in Sweden
Source: JMIR Hum Factors. 2026 Mar 3;13:e75263. doi: 10.2196/75263 (PMC12996901; doi:10.2196/75263)
Supplement: Multimedia Appendix 6 [file humanfactors_v13i1e75263_app6.docx]

**Multimedia Appendix 6 - Informed consent and information about the study**

The version below was translated with DeepL Pro on January 9, 2025. For the original Swedish version, please contact the authors.

**About the SAHA project and this survey**

You are invited to take part in a survey on digital mental health interventions for refugees and migrants. No prior knowledge of this field is required to participate in the survey.

The survey is part of the SAHA research project (<https://liu.se/en/research/saha>) and aims map the knowledge, experience, attitudes and use of digital interventions by health professionals in their work with the target group of refugees.

SAHA is funded by the Swedish Research Council and the survey is conducted by researchers at Linköping University, Mid Sweden University, Stockholm University, and Karolinska Institutet.

**How does the study work?**

If you choose to participate in the study, you will fill out the online questionnaire. The questionnaire is estimated to take about 15-20 minutes. Your participation is anonymous, and your answers will contribute to increased knowledge about how and where interventions for mental health problems in refugees and migrants can be made available in regular care.

**What happens to my data?**

Information provided in the survey will be kept in an encrypted database and stored for at least 10 years after completion of the study. Your answers will be treated confidentially and in anonymized form.

*Click here to find out more about what happens to your data.

**How do I get information about the results of the study?**

Contact one of the study’s contact persons:

Kristofer Vernmark

Licensed psychologist and PhD at Linköping University

E-mail: Kristofer.vernmark@ liu.se

Anahita Geranmayeh

PhD student at Karolinska Institutet

E-mail: anahita.geranmayeh@ ki.se

**Insurance and reimbursement**

No reimbursement is provided. No additional insurance coverage is needed for this study.

**Participation is voluntary**

Your participation is voluntary, and you can choose to stop participating at any time. However, data already collected will be used. If you choose not to participate or wish to withdraw from the study, you do not need to explain why, nor will it affect your situation as a caregiver or you as a professional in your work. If you wish to withdraw your participation, please contact the principal investigator of the study (see below).

**Principal investigator**

Research leader of the SAHA project is Gerhard Andersson

Professor at Linköping University

E-mail: gerhard.andersson@ liu.se

I agree to participate in the survey and that my answers will be collected and processed anonymously by the research team according to GDPR. I am aware that participation is voluntary and that I have the right withdraw at any time without explanation.

Your survey responses are saved - thank you for taking the time to answer this survey!

**What happens to my data?**

Information provided in the survey will be stored in an encrypted database and kept for at least 10 years after the end of the study. Your answers will be treated confidentially and in anonymized form.

*Click here to find out more about what happens to your data.

The results of the study will be presented in scientific journals and conferences. The results will be published at group level and no individual participant will be identified.

Those responsible for handling your personal data in the study are Linköping University and Karolinska Institutet. Your answers and results will be processed in a way that prevents unauthorized access. According to the EU General Data Protection Regulation (GDPR), you have the right to access, free of charge, the data about you that is processed in the study, and to have any errors corrected if necessary.

You can also request that data about you be deleted and that the processing of your personal data be restricted.

If you wish to access or delete the data, please contact the Data Protection Officer who can be reached via email dataskyddsombud@liu.se. If you are dissatisfied with the way your personal data is processed, you have the right to lodge a complaint (see contact details below).

If you are dissatisfied with the way your personal data is processed, you have the right to

lodge a complaint with the Data Protection Authority, which is the supervisory authority.

The project is approved by the Swedish Ethical Review Authority.

**How will I be informed about the results of the study?**

Contact one of the study’s contact persons:

Kristofer Vernmark

Licensed psychologist and PhD at Linköping University

E-mail: [kristofer.vernmark@liu.se](mailto:kristofer.vernmark@liu.se)

Anahita Geranmayeh

PhD student at Karolinska Institutet

E-mail: [anahita.geranmayeh@ki.se](mailto:anahita.geranmayeh@ki.se)

**Insurance and reimbursement**

No reimbursement is provided. No additional insurance coverage is needed for this study.

**Participation is voluntary**

Your participation is voluntary, and you can choose to stop participating at any time. However, data already collected will be used. If you choose not to participate or wish to withdraw, you do not need to give a reason, nor will it affect your situation as a caregiver or you as a professional in your work. If you wish to withdraw your participation, please contact the principal investigator of the study (see below).

**Principal investigator**

Research leader of the SAHA project is Gerhard Andersson

Professor at Linköping University

E-mail: gerhard.andersson@liu.se
