# Supplementary material for: Understanding Psychologists’ Usage, Knowledge, and Attitudes Toward Digital Mental Health Solutions for Refugees and Migrants: Exploratory Cross-Sectional Survey in Sweden
Source: JMIR Hum Factors. 2026 Mar 3;13:e75263. doi: 10.2196/75263 (PMC12996901; doi:10.2196/75263)
Supplement: Multimedia Appendix 7 [file humanfactors_v13i1e75263_app7.docx]

**Multimedia Appendix 7 – Definitions**

This information was provided to participants between informed consent and the first question in the survey. The text below was translated with DeepL Pro on January 9, 2025. For the original Swedish version, please contact the authors.

**Clarification of terms used in the survey**

*About digital assessment/screening and treatment*

We define **digital assessment** as the use of, for example, digital rating scales and other screening tools delivered digitally via smartphone, tablet or computer to detect and assess mental health problems. By **digital treatment** we refer to psychological/psychotherapeutic interventions delivered in a digital format, such as internet-based cognitive behavioral therapy (ICBT), psychological treatment via video/videoconferencing therapy, treatment apps, and treatment via virtual reality (VR).

*Definitions of refugee and migrant*

According to the UN Refugee Convention, a **refugee** is a person who has fled his or her country of origin and is unable to return because of fear of persecution on account of ethnicity, religion, membership of a particular social group or political opinion. A migrant is a person who chooses to move, but not because of a direct threat of persecution or death. Instead, it may be to find a better life by finding work, getting an education, reuniting with family, or for other reasons. When we use the word **migrant** in the survey, we are mainly referring to those migrants who, for various reasons, do not know Swedish well enough to access mental health services in their mother tongue, or prefer to access these services in their mother tongue.
